# Growing together: Developmental integration and modularity in the human talus–calcaneus complex

**DOI:** 10.1111/joa.70186

**Published:** 2026-06-25

**Authors:** Carla Figus, Rita Sorrentino, Francesca Seghi, Maria Giovanna Belcastro, Kristian J. Carlson

**Affiliations:** ^1^ Department of Cultural Heritage University of Bologna, Ravenna Campus Ravenna Italy; ^2^ Department of Biological, Chemical and Pharmaceutical Sciences and Technologies University of Palermo Palermo Italy; ^3^ Department of Biological, Geological and Environmental Sciences – BiGeA University of Bologna Ravenna Italy; ^4^ Division of Integrative Anatomical Sciences Keck School of Medicine, University of Southern California Los Angeles USA; ^5^ Evolutionary Studies Institute University of the Witwatersrand, WITS Johannesburg South Africa

**Keywords:** bipedalism, calcaneus, geometric morphometrics, modularity, morphological integration, ontogeny, talus, trabecular bone

## Abstract

This study investigates age‐related shape changes, morphological integration, and modularity in the human talus–calcaneus complex throughout postnatal development (0–10 years). Geometric morphometric analyses were performed on three‐dimensional landmark data from 23 individuals binned into four age groups, considering both the talus and calcaneus. Statistical analyses included Procrustes ANOVA to assess age effects, two‐block partial least squares to quantify inter‐bone integration, and Covariance Ratio tests to evaluate modular organization within each bone. Trabecular bone architectural parameters from micro‐CT scans were analyzed in parallel. Marked age‐related shape changes were detected in both the talus and calcaneus, with pronounced morphological transitions observed between the middle age groups (1–3 and 3–6 years). The two bones exhibited strong morphological integration across all age classes, representing the first quantitative assessment of talus–calcaneus integration during ontogeny. Modular organization was confirmed also within each bone. Trabecular bone architectural analysis via micro‐CT revealed distinct developmental patterns: the talus exhibited higher initial bone volume fraction with subsequent stabilization and stable trabecular thickness, while the calcaneus showed progressive increases in both bone volume fraction and trabecular thickness, alongside higher anisotropy values, presumably reflecting adaptation to its more direct role in experiencing impact forces during heel strike. The talus–calcaneus complex exhibits highly coordinated ontogenetic timing and strong morphological integration across both external morphology and internal trabecular architecture. These adaptations highlight the sophisticated interplay within this skeletal unit, supporting its function as an integrated biomechanical system critical for understanding human locomotor evolution and foot development.

## Graphical abstract

This study investigates morphological integration and trabecular bone development in the human talus–calcaneus complex during postnatal ontogeny. The two bones exhibit strong integration throughout development, with differing bone volume fraction trajectories presumably reflecting their distinct functional roles during bipedal gait acquisition.

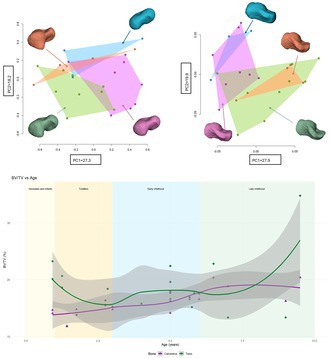

## 1 INTRODUCTION

Human bipedal locomotion represents a defining characteristic of our species, fundamentally distinguishing us from other primates through unique morphological and functional adaptations throughout the skeleton, particularly in the foot bones. Central to the human‐like characteristics of the foot bones is the talocalcaneal complex—comprising the talus and calcaneus forming the hindfoot—which serves as one of the primary bony structural foundations for bipedal locomotion (DeSilva et al., 2019; Harcourt‐Smith & Aiello, 2004; Latimer & Lovejoy, 1989). The morphology of the talocalcaneal complex reflects its critical functional role in human locomotion. The talus, positioned superior to the calcaneus, functions as a sophisticated force transmission center, articulating proximally with the tibia and fibula while forming the subtalar joint distally with the calcaneus. Uniquely among foot bones, the talus lacks direct muscular attachments, enabling efficient force transfer from the leg to the foot without interference from local muscle contractions (Inman, 1976). The calcaneus, as the largest tarsal bone, serves complementary yet distinct functions. It acts as the primary shock absorber for impact forces at heel strike during the gait cycle and provides the insertion point for the Achilles tendon, which generates propulsive forces during push‐off phases of gait. Together, these bones form the subtalar joint complex, which contributes to movements essential for terrain accommodation, propulsion, and balance maintenance during bipedal locomotion (Holowka & Lieberman, 2018; Susman, 1983; Harcourt‐Smith & Aiello, 2004; Elftman & Manter, 1935).

The acquisition of independent locomotion follows a largely predictable developmental sequence that progressively challenges the talocalcaneal complex. A critical locomotor event occurs between 8 and 18 months when infants progress from assisted to independent walking (Karasik et al., 2012, 2015; Sutherland et al., 1980), altering mechanical loading and stimulating morphological changes in the talocalcaneal complex (Figus et al., 2022, 2025; Figus, Sorrentino, et al., 2023).

Early walkers display a wide base of support, short stride length, flat foot contact, and high hip lift (Hallemans et al., 2006; Sutherland, 1997; Zeininger et al., 2018). With increasing locomotor experience, children develop more refined gait features and greater consistency in gait parameters, including plantarflexion at toe‐off and heel strike at initial contact. Effective heel strike is established between 18 and 24 months (Bertsch et al., 2004; Sutherland et al., 1980), and by approximately 2 years of age, children adopt an adult‐like knee flexion‐extension pattern (Zeininger et al., 2018). The longitudinal arch begins forming with the onset of bipedal locomotion but only reaches functional maturity between ages 4 and 6 years (Bertsch et al., 2004; Zeininger et al., 2018). Although most kinematic aspects of gait have reached mature states by this stage, muscle activation patterns continue to evolve and do not fully resemble adult profiles until adolescence (Sutherland, 1997; Sutherland et al., 1980).

From an evolutionary perspective, the talocalcaneal complex provides critical insights into the origins and refinement of human bipedalism. Unique morphological features of the talus and calcaneus that develop during ontogeny—including the robust calcaneal tuberosity, the medially oriented talar neck, and the expanded talar trochlea—distinguish humans from other primates and reflect selective pressures linked to habitual terrestrial bipedalism (Lovejoy, 1988; Susman, 1983). Fossil evidence from early hominins reveals progressive changes in talar and calcaneal morphology corresponding to increasing reliance on terrestrial bipedal locomotion, positioning these bones as key indicators of locomotor strategies across hominin taxa (Ward et al., 2011; Zipfel et al., 2011).

### 1.1 State‐of‐the‐art

Geometric morphometric studies of the talus and calcaneus have significantly advanced our understanding of shape variation within both paleoanthropological and anthropological contexts. Research on the talus has explored its evolutionary trajectory and functional morphology, including analyses of extinct hominins (Gebo, 1992; Gebo & Schwartz, 2006; Latimer et al., 1987; Lovejoy et al., 2009; Sorrentino, Carlson, et al., 2020), as well as climbing likelihood in early hominins (DeSilva, 2009; Prang et al., 2025; Venkataraman et al., 2013).

Similarly, extensive morphometric work has been conducted on the calcaneus, with foundational studies by Latimer and Lovejoy (1989) establishing patterns of calcaneal shape variation relevant to bipedalism. Subsequent studies examined calcaneal robusticity, morphology across diverse primate taxa (Raichlen et al., 2010), comprehensive shape analyses (Harper et al., 2021a, 2021b), and early hominin morphology (Prang, 2016; Zipfel et al., 2011; Zipfel & Berger, 2009). Recent work has explored calcaneal tuber morphology and its links to heel strike mechanics and endurance running (Holowka et al., 2017; Holowka & Lieberman, 2018; Prang, 2023).

Beyond external morphology, trabecular bone analysis has significantly expanded understanding of internal bone structure within the talocalcaneal complex. Tsegai et al. (2013, 2017, 2018) demonstrated how developmental trajectories in talar trabecular architecture correlate with loading patterns in both modern and fossil hominins, mirroring changes in gait mechanics during growth. Parallel investigations on the calcaneus by Saers and colleagues (Saers, 2023; Saers et al., 2020, 2022) have examined trabecular organization and bone volume fraction across developmental stages, revealing internal adaptations to varying mechanical stresses related to foot strike patterns and load transmission strategies. Comparative trabecular analyses have also contributed to understanding locomotor evolution in fossil hominins and across extant primate taxa (Chirchir et al., 2015; Ryan et al., 2018; Ryan & Krovitz, 2006; Ryan & Shaw, 2012, 2015; Su & Carlson, 2017; Tsegai et al., 2017, 2018), providing insights into the mechanical adaptations distinguishing human bipedalism from other locomotor modes.

Contemporary paleoanthropological and biological anthropological research increasingly employs theoretical frameworks of morphological integration and modularity to interpret shape variation within the foot skeleton. Morphological integration describes the coordinated variation among interconnected anatomical structures that function together, while modularity refers to the relative independence of certain skeletal units that can vary semi‐autonomously (Klingenberg, 2013; Klingenberg, 2016). Morphological integration posits that interconnected skeletal elements exhibit coordinated shape changes to preserve joint function (Adams, 2016; Klingenberg, 2013). However, despite these theoretical advances, studies specifically examining patterns of covariation between the talus and calcaneus during growth remain scarce, leaving important questions about the developmental integration of the talocalcaneal complex unanswered.

Ontogenetic changes within individual bones of the talocalcaneal complex have been investigated (Figus et al., 2022; Figus, Sorrentino, et al., 2023; Figus, Stephens, et al., 2023; Saers et al., 2020, 2022). These studies have demonstrated that both external morphology and internal trabecular architecture change during growth, reflecting adaptations to increasing mechanical demands during locomotor development. However, these investigations primarily focused on individual bones rather than examining how the talus and calcaneus develop in coordination with each other or how they may show patterns of morphological integration throughout ontogeny. How these two functionally integrated elements covary during development therefore remains insufficiently explored. By combining external shape analysis with integration and modularity tests, this study seeks to advance current understanding of human hindfoot development.

### 1.2 Objectives and hypotheses

This study undertakes a comprehensive and exploratory analysis of morphological integration and modular organization in the developing talus–calcaneus complex. Our previous studies examined the talus and calcaneus separately, documenting significant age‐related shape changes in each bone independently (Figus et al., 2022, 2025; Figus, Stephens, et al., 2023). However, those analyses could not assess how these two functionally interconnected bones covary during development. Here, we address this gap by analyzing a subset of 23 individuals (ages 0–10 years) for whom both the talus and calcaneus are preserved, allowing us to test patterns of integration and modularity between the two bones. Specifically, we test two hypotheses:

- **H1 (morphological integration)**: The talus and calcaneus exhibit strong morphological integration in external shape throughout ontogeny, reflecting their biomechanical interdependence within the subtalar joint complex. Based on their articular relationship and shared functional role, this hypothesis is grounded in coordinated growth patterns and maintained joint congruence (Hellier & Jeffery, 2006; Saers et al., 2020). We predict that shape changes in one bone will covary with complementary changes in the other during the course of development.
- **H2 (modular organization):** Despite strong inter‐bone integration, anatomical modularity within each bone allows distinct subregions of external shape to adapt semi‐independently to localized mechanical stresses during growth. This hypothesis is supported by general principles of skeletal modularity (Mitteroecker & Bookstein, 2009) and preliminary evidence from talocalcaneal studies (Saers et al., 2020). We predict that functionally distinct regions within each bone (e.g., articular surfaces vs. tuberosities) will exhibit modular organization, while the bones as a whole maintain coordinated shape variation with each other.

## 2 MATERIALS AND METHODS

### 2.1 Sample

This study includes 23 juvenile individuals (Table S1) with both the talus and calcaneus preserved, enabling paired morphometric analyses of external shape. This sample is a subset of individuals analyzed in Figus et al. (2022, 2025), selected to allow direct comparisons between the talus and calcaneus. Ethical approvals and access permissions were obtained from all curating institutions (Department of Biological, Geological and Environmental Sciences – BiGeA, University of Bologna; Superintendence of Archaeology, Fine Arts and Landscape for the provinces of Salerno and Avellino), and all research followed ethical guidelines for the study of human skeletal remains as outlined by the American Association of Biological Anthropologists. The inclusion of both modern documented and archaeological individuals was motivated by the need to capture a broader spectrum of developmental variation across different environmental and temporal contexts. This design allows an exploratory assessment of whether patterns of talus–calcaneus integration and modularity are consistent across heterogeneous populations and preservation conditions.

The sample spans a developmental range of approximately 0.5 to 10 years and includes specimens from both modern documented collections and archaeological contexts (Table S1):

- Bologna (Italy, modern documented collection, 20th century): 14 individuals (6 males, 8 females), with known sex and age‐at‐death information derived from archival records (Belcastro et al., 2017, 2025; Sorrentino et al., 2025). Ages range from approximately 0.9 to 9 years.
- Velia (Italy, Imperial Roman period, 1st century BCE – 3rd century CE): 9 individuals aged approximately 0.5 to 9.5 years; sex undetermined. Age estimates are based on standard osteological methods including dental development and long bone metrics (Bondioli et al., 2016; [ref Velia])

Individuals were grouped into four developmental stages corresponding to major locomotor transitions during the first decade of life: 0–1 years (pre‐walking), 1–3 years (independent walking), 3–6 years (heel‐strike and arch maturation), and 6–10 years (mature gait) (Bertsch et al., 2004; Sutherland et al., 1980; Zeininger et al., 2018; Figus et al., 2025; Swan et al., 2020):

- 0–1 years (Neonates and infants): 3 individuals (~6–11 months)
- 1.1–3 years (Toddlers): 11 individuals (~1.25–3 years)
- 3.1–6 years (Early childhood): 4 individuals (~4–6 years)
- 6.1–10 years (Late childhood): 6 individuals (~6.5–9.5 years)

### 2.2 Data acquisition

This study utilizes three‐dimensional landmark coordinates and trabecular bone architectural data from left tali and calcanei previously analyzed in Figus et al. (2022, 2025). All specimens were scanned using high‐resolution computed tomography with voxel resolutions ranging from 12 to 38 μm. Complete CT acquisition parameters, segmentation protocols, and trabecular quantification methods are described in Figus et al. (2022, 2025).

### 2.3 Geometric morphometric analysis

**FIGURE 1:**
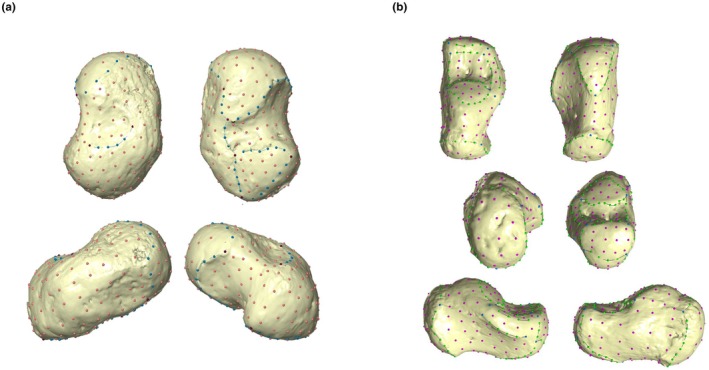
Landmark and semilandmark configurations. (a) Talus template (modified from Figus et al., 2025): Top row (left to right): superior and inferior views; bottom row (left to right): medial and lateral views. Magenta spheres = fixed anatomical landmarks; cyan/blue spheres = curve semilandmarks; green spheres = surface semilandmarks. (b) Calcaneus template (modified from Figus et al., 2022): Top row (left to right): superior and inferior views; middle row (left to right): posterior and anterior views; bottom row (left to right): medial and lateral views. Red/magenta spheres = fixed anatomical landmarks; green spheres = curve semilandmarks; yellow/green spheres = surface semilandmarks. For complete landmark definitions, anatomical descriptions, and placement protocols, see Figus et al. (2022) for the calcaneus and Figus et al. (2025) for the talus.

Geometric morphometric analysis was previously performed on the external morphology of both the talus and calcaneus in Figus et al. (2022, 2025), using the landmark templates illustrated in Figure 1:

- Calcaneus template (Figus et al., 2022): 180 landmarks and semilandmarks, consisting of 15 fixed anatomical landmarks, 88 curve semilandmarks, and 77 surface semilandmarks.
- Talus template (Figus et al., 2025): 228 landmarks and semilandmarks, consisting of 8 fixed anatomical landmarks, 45 curve semilandmarks, and 175 surface semilandmarks.

The coordinates generated in those studies were used for the present integration and modularity analyses. Three‐dimensional landmarks and semilandmarks coordinates were collected using Viewbox 4 (dHAL Software). Sliding semilandmarks were allowed to slide along curves or across surfaces to minimize bending energy, ensuring optimal geometric homology across specimens (Gunz et al., 2005; Mitteroecker & Bookstein, 2007). All landmarks configurations were aligned using Generalized Procrustes Analysis (GPA) to remove non‐shape variation due to size, position, and orientation (Rohlf & Slice, 1990). Centroid size was computed for each specimen as the square root of the sum of squared distances of all landmarks from their centroid, serving as a proxy for overall bone size.

Modules correspond to anatomically and functionally distinct regions of the talus and calcaneus, defined a priori based on established morphological and biomechanical knowledge of the talocalcaneal complex. These regions reflect coherent structural units involved in articulation and load transmission (e.g., articular facets and supporting elements) and were delineated to capture functionally meaningful subdivisions of each bone. Module definition is therefore hypothesis‐driven and grounded in prior anatomical understanding rather than data‐driven partitioning. All regions were defined to be consistently identifiable across the full ontogenetic range analyzed, despite ongoing morphological change during growth.

- Talar modules: Head (53); Posterior calcaneal facet (28); Trochlea (37).
- Calcaneal modules: Sustentaculum tali (15); Posterior talar facet (35).

### 2.4 Statistical analyses

A principal component analysis was run in R (R Core Team. R: A Language and Environment for Statistical Computing. Vienna, Austria, 2025, v. 4.5.0). Procrustes ANOVAs were conducted to evaluate the extent to which shape variation is explained by age classes (whole sample), sex (Bologna sample only), and chronological period. When significant effects were detected, pairwise comparisons of Procrustes distances between age groups were performed to identify specific shape differences. Allometric effects were assessed by multivariate regression of shape variables on the natural logarithm of centroid size (lnCS) using the geomorph R package (v. 4.0.10; Adams & Collyer, 2009, 2018). To test whether allometric trajectories differ among age groups, an interaction model (Age Class × lnCS) was fitted.

Shape variation related to age was modeled using two complementary approaches in the Procrustes ANOVA. Firstly, age was treated as a categorical variable (four age classes: 0–1, 1–3, 3–6, and 6–10 years) to specifically test for significant shape differences between groups corresponding to major locomotor and functional developmental milestones (e.g., walking acquisition). Secondly, age was included as a continuous variable to capture and quantify the overall smooth developmental trajectory across the entire postnatal range, thereby providing a comprehensive view of ontogenetic change. The use of both categorical and continuous models ensured that age effects were assessed at both discrete functional transition points and across the entire developmental period.

To quantify morphological integration between the talus and calcaneus, two‐block partial least squares (2B‐PLS) analysis was performed using the *integration.test* function in the geomorph R package (v. 4.0.10; Adams & Collyer, 2018). The strength of integration was assessed using the PLS correlation coefficient (r‐PLS) and effect size (*Z*‐score), while significance was tested via permutation. Integration between predefined anatomical modules within each bone was assessed using the same approach.

Modularity was tested using the Covariance Ratio (CR) via the *modularity.test* function in the *geomorph* R package (v. 4.0.10), which compares the covariance among landmarks within modules to the covariance between modules. CR is based on covariance structure and evaluated through permutation procedures and is generally considered relatively robust to differences in sample size compared to alternative modularity metrics. A CR value lower than expected under the null hypothesis of no modularity indicates the presence of modular structure. Statistical significance was assessed via permutation testing (10,000 iterations).

All statistical analyses employed Residual Randomization in Permutation Procedures (RRPP) with 999 permutations to assess statistical significance, based on Ordinary Least Squares (OLS) regression models using the RRPP package v. 2.1.2 (Collyer & Adams, 2024, 2018). In line with recent discussions on statistical inference (e.g., Courtenay, 2024), *p*‐values are interpreted cautiously and in conjunction with effect sizes and permutation‐based evidence rather than strict binary thresholds.

Trabecular bone microstructural parameters were analyzed for a subsample of tali (*n* = 22) and calcanei (*n* = 14). Raw data for key microarchitectural parameters—including bone volume fraction (BV/TV), trabecular thickness (Tb.Th), trabecular number (Tb.N), and degree of anisotropy (DA)—were obtained from and further analyzed based on methodologies described in Figus et al. (2022, 2025).

To visualize the developmental trajectories of these parameters as a function of age, we employed Locally Estimated Scatterplot Smoothing (LOESS) regression. A LOESS curve, with a smoothing span set to 0.75, was fitted to the scatterplot data for each microstructural parameter against continuous age to illustrate non‐linear developmental trends.

## 3 RESULTS

### 3.1 External morphology

#### 3.1.1 Principal component analysis

**FIGURE 2:**
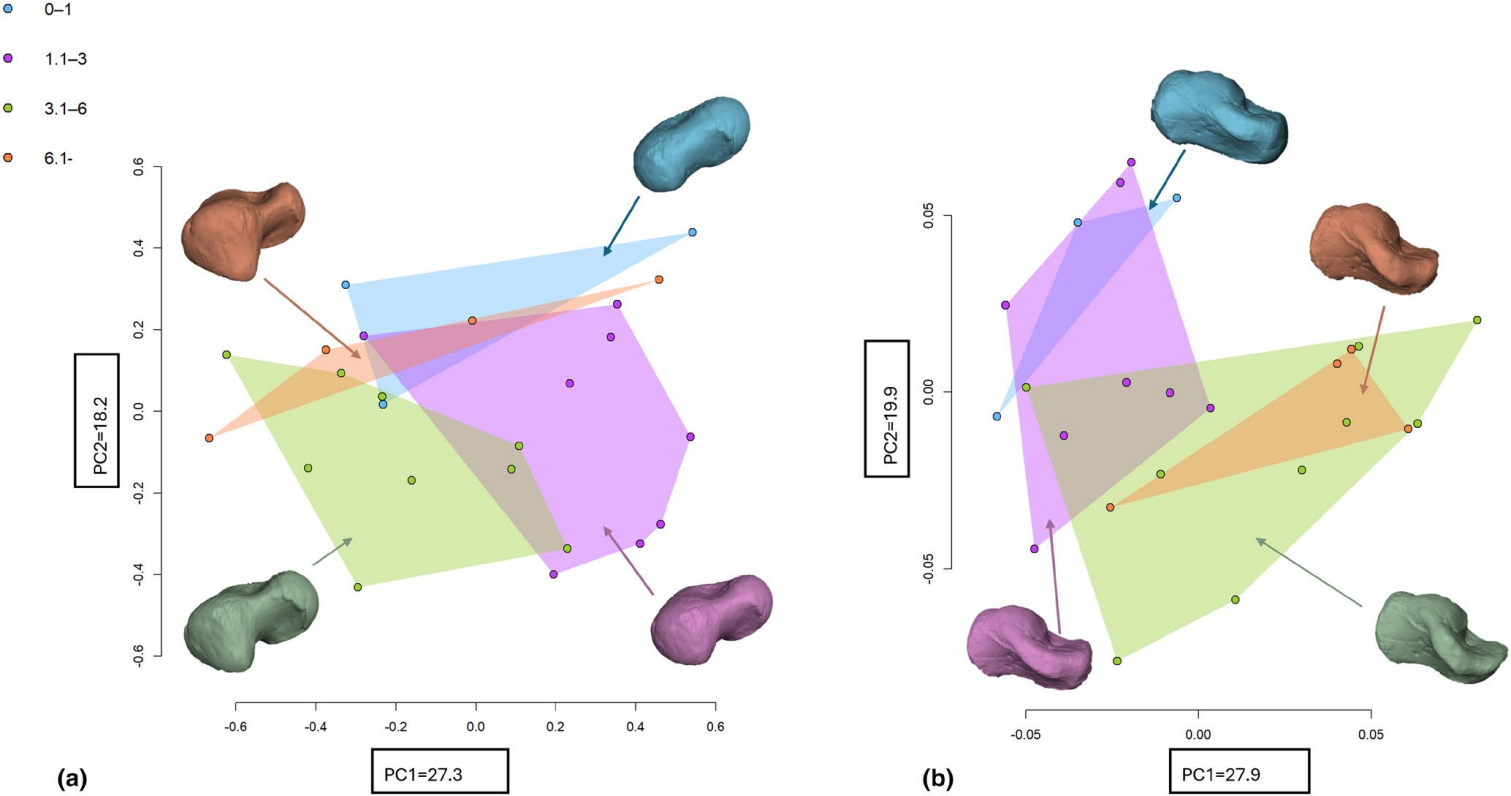
Principal component analysis of talar and calcaneal shape variation during ontogeny. Scatterplots show specimen distribution in morphospace defined by PC1 and PC2 for (a) talus and (b) calcaneus. Specimens are color‐coded by age class: blue = 0–1 years; purple = 1–3 years; yellow = 3–6 years; brown = 6–10 years. Colored polygons encompass each age group; 3D meshes represent mean group shapes.

Principal component analysis (PCA) of shape variation revealed that the first three components accounted for a substantial proportion of total variance in both bones (Figure 2). For the talus, PC1 explained 27.3%, PC2 18.2%, and PC3 10.6%, totaling 56.1% of variance. For the calcaneus, PC1 explained 27.9%, PC2 19.9%, and PC3 11.1%, totaling 57.7% of variance.

Morphologically, PC1 in the talus represents the primary axis of ontogenetic change, reflecting the reorientation of the talar neck (shorter in the youngest individuals, described by negative scores, to being elongated, more medially oriented in the oldest individuals, described by positive scores) and correlated changes in the head. PC2 reflects variation in the curvature of the trochlea and the posterior calcaneal facet. In the calcaneus, PC1 captures the developmental shift in overall body shape and robusticity, specifically the increased length and definition of the posterior area of the body and a change in the relative size of the sustentaculum tali. PC2 primarily reflects variation in the orientation and robusticity of the sustentaculum tali and the overall curvature of the calcaneal body.

Significant ontogenetic shape changes were observed in both the talus and calcaneus. When age was modeled as a categorical variable (four age classes), it significantly influenced shape variation in the talus (*F* = 2.33, *df* = 3, *R* ^2^ = 0.26, *p* < 0.001) and the calcaneus (*F* = 2.36, *df* = 3, *R* ^2^ = 0.27, *p* = 0.001). When age was treated as a continuous variable, it remained a significant predictor of talar shape (*F* = 5.88, *df* = 1, *R* ^2^ = 0.21, *p* = 0.001) and calcaneal shape (*F* = 4.70, *df* = 1, *R* ^2^ = 0.18, *p* = 0.001). Across both model specifications, results were consistent, indicating a robust ontogenetic signal in shape variation regardless of how age is modeled.

Pairwise comparisons between age groups identified shape differences between groups (Table 1). The most pronounced morphological differences occurred between the 1–3‐year and 3–6‐year groups in both bones (talus: *d* = 0.09, *p* = 0.004; calcaneus: *d* = 0.06, *p* = 0.008), representing the largest change among consecutive age groups. Comparatively strong differences were also observed in the youngest (0–1 years) and oldest (6–10 years) age groups for both the talus (*d* = 0.12, *p* = 0.02) and calcaneus (d = 0.09, *p* = 0.009). No detectable differences were found between the 3–6‐ and 6–10‐year groups.

**TABLE 1:** Pairwise procrustes distances and significance levels between age groups.

Age group comparison	Talus		Calcaneus	
	*d*	*p*‐value	*d*	*p*‐value
0–1 versus 1–3	0.054	0.940	0.062	0.249
0–1 versus 3–6	0.095	0.065	0.085	**0.005**
0–1 versus 6–10	0.122	0.020	0.096	**0.009**
1–3 versus 3–6	0.094	0.004	0.066	**0.008**
1–3 versus 6–10	0.116	0.002	0.073	**0.036**
3–6 versus 6–10	0.066	0.390	0.046	0.499

*Note*: Bold indicates *p* < 0.05. The 1–3 to 3–6 year transition represents the largest morphological change between consecutive age groups in both bones.

Abbreviation: *d*, Procrustes distance.

#### 3.1.2 Allometric effects and sex and diachronic variation

Allometric effects were found in both bones (Table 2), with shape variation accounted for by *R* ^2^ = 0.219 in the talus (*F* = 5.88, *df* = 1.21, *p* = 0.001) and *R* ^2^ = 0.202 in the calcaneus (*F* = 5.31, *df* = 1.21, *p* = 0.001). Tests for an interaction between age class and size revealed no evident effect for the talus (*F* = 0.74, *df* = 3.15, *p* = 0.862), suggesting consistent allometric patterns across age groups. For the calcaneus (*F* = 1.55, *df* = 3.15, *p* = 0.049), results suggest subtle differences in size–shape relationships across developmental stages.

**TABLE 2:** Procrustes ANOVA results for allometry and age × size interaction.

Analysis	Bone	Effect	*df*	*F*	*R* ^2^	*p*‐value
Allometry (shape ~ size)	Talus	log(centroid size)	1. 21	5.88	0.219	**0.001**
	Calcaneus	log(centroid size)	1. 21	5.31	0.202	**0.001**
Age × size interaction	Talus	Age class × log(size)	3. 15	0.74	0.086	0.862
	Calcaneus	Age class × log(size)	3. 15	1.55	0.160	0.049

*Note*: Bold indicates *p* < 0.05.

Sex, evaluated only for the modern collection, did not influence talar (*F* = 0.54, *df* = 1, *R* ^2^ = 0.043, *p* = 0.900) or calcaneal shape variation (*F* = 0.57, *df* = 1, *R* ^2^ = 0.046, *p* = 0.868). Chronological period (modern vs. archaeological specimens) also did not show a detectable effect on talar shape (*F* = 1.37, *df* = 1, *R* ^2^ = 0.061, *p* = 0.168), whereas differences in calcaneal shape were observed on calcaneal shape (*F* = 2.37, *df* = 1, *R* ^2^ = 0.102, *p* = 0.025). All Procrustes ANOVA results are summarized in Table 3.

**TABLE 3:** Procrustes ANOVA results for shape variation in talus and calcaneus.

Effect tested	Bone	*df*	*F*	*R* ^2^	*p*‐value
Age class	Talus	3	2.33	0.269	**0.001**
	Calcaneus	3	2.36	0.271	**0.001**
Chronological period	Talus	1	1.37	0.061	0.168
	Calcaneus	1	2.37	0.102	**0.025**
Sex	Talus	1	0.54	0.043	0.900
	Calcaneus	1	0.57	0.046	0.868

*Note*: Bold indicates *p* < 0.05.

#### 3.1.3 Morphological integration

Morphological integration between the talus and calcaneus was high (r‐PLS = 0.89, *p* < 0.001, Figure S1), supported by a large effect size (*Z* = 3.10), as demonstrated by Two‐block partial least squares (2B‐PLS) analysis. This indicates that shape changes in one bone during growth are strongly associated with shape changes in the other, reflecting their functional interdependence within the talocalcaneal joint complex.

Integration between directly articulating surfaces was also confirmed (Figure S2). Specifically, strong covariation was observed between the posterior calcaneal facet (talus) and the posterior talar facet (calcaneus) (r‐PLS = 0.878, *Z* = 3.09, *p* < 0.001), supporting their direct functional relationship as components of the subtalar joint. This pattern of integration was consistent across the entire age range examined (0–6 years).

Clear modular organization was detected within each bone using Covariance Ratio (CR) analysis. The talus exhibited modular organization (CR = 0.842, *p* < 0.001, effect size = −15.37), with distinct modules including the head, posterior calcaneal facet, and the trochlea. The calcaneus similarly showed a modular architecture (CR = 0.817, *p* < 0.001, effect size = −11.37), with the sustentaculum tali and the posterior talar facet forming distinct modules.

Despite this modular organization, substantial inter‐module integration was observed within both bones. The full results for inter‐bone and intra‐module integration are detailed in Table 4.

**TABLE 4:** Partial least squares (PLS) integration results for the talus–calcaneus complex.

Comparison (modules/blocks)	Bone/complex	Correlation (*r*‐PLS)	*Z* (effect size)	*p*‐value
Talus versus calcaneus integration (overall)	Inter‐bone (general)	0.89	3.10	**0.001**
Posterior calcaneal facet (talus) versus posterior talar facet (calcaneus)	Inter‐bone (articular)	0.878	3.09	**0.001**
Head versus posterior calcaneal facet	Intra‐talus	0.877	3.36	**0.001**
Head versus trochlea	Intra‐talus	0.862	3.27	**0.001**
Posterior calcaneal facet versus trochlea	Intra‐talus	0.881	3.54	**0.001**
Sustentaculum tali versus other	Intra‐calcaneus	0.900	3.49	**0.001**
Posterior talar facet versus other	Intra‐calcaneus	0.897	3.60	**0.001**

*Note*: Bold indicates *p* < 0.05.

### 3.2 Internal morphology

**FIGURE 3:**
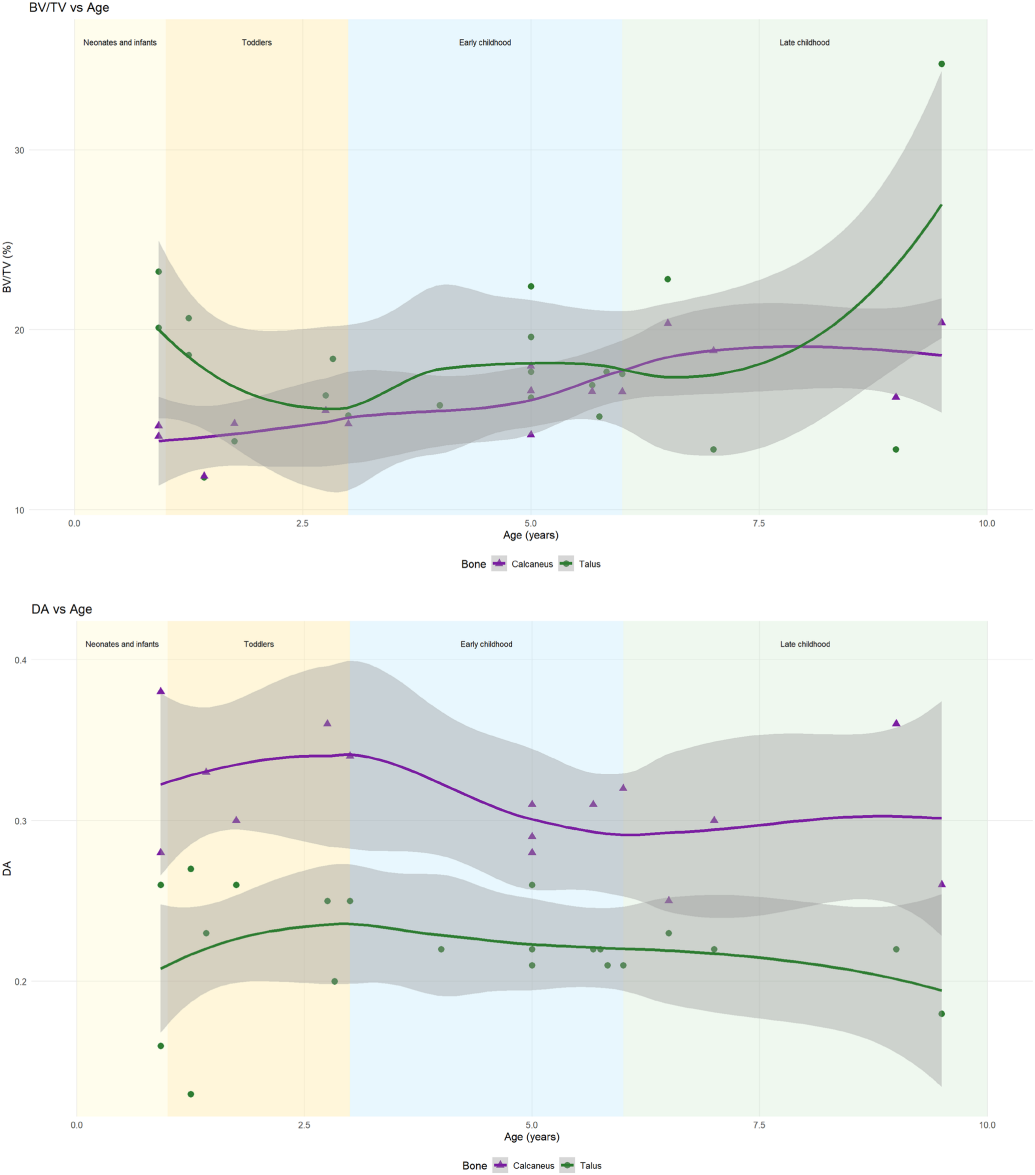
Trabecular bone developmental patterns in the talus and calcaneus. (Top) Bone volume fraction (BV/TV) by continuous age for both bones. (Bottom) Degree of anisotropy (DA) by continuous age for both bones. Background shading indicates the four developmental phases based on locomotor milestones: Neonates and infants (pale yellow, 0–1 years); toddlers (light orange, 1–3 years, walking acquisition); early childhood (light blue, 3–6 years, mature heel‐strike and arch formation); late childhood (light green, 6–10 years, mature gait patterns). Green circles = talus; purple triangles = calcaneus. Lines represent locally weighted regression (LOESS) smoothing (span = 0.75) to visualize trends. Sample sizes: talus *n* = 22, calcaneus *n* = 14.

Trabecular bone microarchitecture parameters show clear developmental patterns during early childhood (Tables 5 and 6 and S2, Figure 3). Due to differential preservation, fewer calcanei were available for microstructural analysis (*n* = 14) compared to tali (*n* = 22), though developmental trajectories in each bone remain evident across the available age range.

Both bones exhibited characteristic trabecular (re)modeling with increasing age: reductions in trabecular number (Tb.N), increases in trabecular thickness (Tb.Th), and increases in trabecular separation (Tb.Sp) (Table 2). This combination of trends reflects the ontogenetic change from a dense network of thin trabeculae in infancy to a sparser network of thicker, more robust trabeculae by late childhood.

Bone volume fraction (BV/TV) and degree of anisotropy (DA) revealed distinct patterns between the two bones (Tables 5 and 6). The talus maintained relatively stable BV/TV (~17%–21%) and DA (~0.22) throughout development. In contrast, the calcaneus showed progressive increases in BV/TV (from ~12% to ~20%) and consistently higher anisotropy values (DA ~0.28–0.36).

**TABLE 5:** Trabecular architectural parameters by age group (mean ± SD).

Age group (years)	Bone	*n*	Tb.N (mm^−1^)	Tb.Th (mm)	Tb.Sp (mm)
0–1	Talus	3	1.43 ± 0.08	0.15 ± 0.02	0.55 ± 0.06
	Calcaneus	2	1.09 ± 0.17	0.17 ± 0.02	0.74 ± 0.17
1–3	Talus	7	1.21 ± 0.07	0.18 ± 0.03	0.65 ± 0.06
	Calcaneus	4	0.94 ± 0.09	0.46 ± 0.08	3.01 ± 0.91
3.1–6	Talus	4	1.20 ± 0.14	0.20 ± 0.02	0.64 ± 0.08
	Calcaneus	2	0.90 ± 0.03	0.50 ± 0.04	3.90 ± 0.35
6.1–10	Talus	6	1.16 ± 0.08	0.23 ± 0.05	0.62 ± 0.05
	Calcaneus	6	0.80 ± 0.14	0.66 ± 0.12	5.43 ± 1.63

Abbreviations: *n*, number of individuals with trabecular data; Tb.N, trabecular number; Tb.Sp, trabecular separation; Tb.Th, trabecular thickness.

**TABLE 6:** Bone volume fraction and anisotropy by age group (mean ± SD).

Age group (years)	Bone	*n*	BV/TV (%)	DA
0–1	Talus	3	21.16 ± 1.65	0.23 ± 0.07
	Calcaneus	2	14.38 ± 0.41	0.33 ± 0.05
1–3	Talus	7	16.41 ± 3.26	0.23 ± 0.04
	Calcaneus	4	14.24 ± 1.70	0.33 ± 0.03
3.1–6	Talus	4	18.73 ± 3.12	0.23 ± 0.02
	Calcaneus	2	15.89 ± 1.75	0.31 ± 0.03
6.1–10	Talus	6	17.14 ± 6.82	0.21 ± 0.02
	Calcaneus	6	19.23 ± 1.98	0.29 ± 0.05

*Note*: Total sample: talus *n* = 22, calcaneus *n* = 14 due to differential preservation. Sample sizes differ from shape analysis (*n* = 23 total) as not all specimens had sufficient preservation for trabecular quantification.

Abbreviations: BV/TV, bone volume fraction; DA, degree of anisotropy; *n*, number of individuals with trabecular data.

## 4 DISCUSSION

### 4.1 Ontogenetic shape changes and allometric independence

Geometric morphometric analyses revealed pronounced ontogenetic shape changes in both the talus and calcaneus. Age accounted for substantial variance in shape for both bones when modeled categorically and remained a strong predictor when treated as a continuous variable, indicating consistent trends throughout development. The higher variance explained by categorical models of age suggests that ontogenetic changes are more clearly detectable using distinct age phases rather than modeling age as a continuous gradual process.

Pairwise comparisons following Procrustes ANOVA identified the 1–3 to 3–6‐year period as showing the most pronounced differences between consecutive age groups in both bones, alongside the generally pronounced differences between the youngest and oldest age groups. In the talus, these changes involve increasing medial orientation of the head and neck, expansion and curvature of the trochlear surface, and better definition of articular facet margins (Figus et al., 2022, 2023). In the calcaneus, key changes include medial expansion of the sustentaculum tali, thickening of the calcaneal tubercle, more vertical orientation of the posterior talar facet, and maturation of the distal configuration with better‐defined anterior talar and cuboid facets (Figus et al., 2025). These coordinated changes suggest an integrated developmental response where both bones expand their articular surfaces medially (widening the base of support), improve joint congruence (stabilizing the talocalcaneal complex), and reorient load‐bearing surfaces (accommodating mature compressive loading patterns). These morphological changes are prerequisites for maintaining a functional longitudinal arch. This critical change around year 3 coincides temporally with key biomechanical developments that characterize bipedal gait acquisition: children adopt adult‐like knee flexion patterns by 2 years (Zeininger et al., 2018), and the longitudinal arch reaches functional maturity between 4 and 6 years (Bertsch et al., 2004; Zeininger et al., 2018).

Trabecular data further support this critical developmental window, with both bones showing characteristic (re)modeling patterns including reductions in trabecular number and increases in trabecular thickness and separation during this period. Our morphometric data suggest that a coordinated structural reorganization takes place during this 2–4‐year window, adapting the talus and calcaneus externally and internally to accommodate the progressive changes occurring in loading patterns. The magnitude of shape differences between 1–3‐year and 3–6‐year groups exceeds that of any of the other consecutive age group pairings in the study, underscoring this period as a critical phase of hindfoot functional maturation.

During the 1–3‐year period, as children acquire independent walking proficiency, the observed morphological shifts likely stem from bone plasticity responding to increasing mechanical stresses associated with gait refinement, including the emergence of a distinct toe‐off mechanism and longitudinal arch maturation. Increased stress magnitudes result both from growth‐related mass increases and from higher walking speeds as gait becomes more stable (Sutherland et al., 1980). This early locomotor phase introduces dynamic loading patterns that are distinct from the relatively passive mechanical loading that occurs during supported standing (Raichlen et al., 2015). Our sample, beginning at approximately 6–11 months of age, captures individuals both before and after the onset of independent walking, revealing morphological differences in talar neck orientation and calcaneal tuberosity robusticity between pre‐walking infants and toddlers with established gait. While prior research detailed talar adaptations for optimizing force transmission and joint mobility (Figus et al., 2022), our current integrated analysis of the talus–calcaneus complex reveals that both bones undergo simultaneous substantial structural modifications for accommodating enhanced shock absorption associated with the ground reaction forces of substrate contact. This coordinated response highlights that hindfoot functional maturation is a systemic process, whereby distinct morphological adaptations in each element synergistically contribute to achieving overall gait efficiency and stability. Subsequent changes from 3 to 6 years may reflect further optimization as gait kinematics become more consistent and stereotyped, alongside accommodation of greater stability challenges associated with increased speeds, varied substrate usage, and more complex locomotor activities such as running and jumping, manifested in our data as subtle refinement of the subtalar articular surfaces and increased robusticity of the calcaneal posterior tubercle.

Allometric effects were found in both bones, indicating that size‐related shape changes contribute substantially to overall morphological variation in the sample. However, the absence of detectable age‐size interactions for the talus and a marginally detectable interaction for the calcaneus suggest that the observed morphological changes represent active functional adaptation rather than mere passive scaling effects. The lack of interaction in the talus indicates that the relationship between size and shape remains consistent across age groups for this bone; shape changes associated with age occur independently of those driven by size increase. This pattern suggests that shape modifications are specifically tailored to changing functional demands of developing bipedal gait rather than being simple byproducts of growth‐related size increases (Klingenberg, 2016).

Based on these results, shape changes appear to be driven primarily by the mechanical demands of locomotion in combination with allometric effects, rather than by size increase alone. Morphological modifications in the talus likely are responses to its primary role in transmitting body weight from the leg to the foot and to complex, multi‐directional joint movements crucial for balance and propulsion. Concurrent changes in the calcaneus reflect responses to its function in ground contact and shock absorption as gait patterns mature. As children transition from unstable toddler gait to more refined locomotion, both the magnitude and consistency of loading patterns change, requiring structural adaptations beyond those predicted by size scaling alone (Raichlen et al., 2015; Saers et al., 2020).

Sex was not a strong factor in talar or calcaneal shape variation, confirming that sexual dimorphism is limited during childhood. This likely reflects the similar activity levels and growth trajectories of girls and boys during early development, before the onset of puberty‐related divergence. Age‐related patterns appeared consistent across specimens regardless of temporal origin or geographic provenance, although the limited sample size and heterogeneous composition warrant caution in generalizing these developmental findings. Future studies with larger samples spanning multiple populations and extended temporal ranges could investigate potential population‐level or environmental influences on hindfoot development.

### 4.2 Morphological integration and modular organization

Our analyses revealed strong morphological integration of the talus and calcaneus, demonstrating this pair of skeletal elements is a highly coordinated functional unit. Shape changes in one bone are highly predictable from shape changes in the other, reflecting biomechanical interdependence within the hindfoot complex. This integration aligns with their anatomical and functional relationships, enabling coordinated hindfoot motion (e.g., inversion and eversion) that promotes locomotor stability on variable terrain (Nester et al., 2007). The observed level of integration is notably higher than typically reported for functionally related skeletal elements in other anatomical regions (such as elements of the hand or the cranial vault, for example, Goswami, 2006; Sardi et al., 2011), suggesting that the biomechanical demands of bipedal locomotion impose particularly strong constraints on coordinated development in these hindfoot elements. Integration patterns were consistent across our archaeological and modern samples, suggesting that these developmental constraints may be relatively robust to environmental differences between populations (e.g., footwear use). However, this preliminary observation requires confirmation with larger, more diverse samples. In addition, differential preservation may have affected the composition of the archaeological subsample, particularly for trabecular analyses, and potential preservation‐related biases should therefore be considered when interpreting these results. This robust integration represents a key adaptation for obligate bipedalism, as disruptions in coordinated talocalcaneal development can lead to biomechanical inefficiencies, joint incongruency, and pathological conditions (e.g., tarsal coalition, severe pes planus, and subsequent subtalar arthritis; Sarrafian, 2011; Mosca, 2015), implying strong selective pressures to maintain this integrated developmental unit throughout ontogeny.

Despite strong overall developmental integration, both the talus and calcaneus also exhibit clear modular organization as semi‐independent anatomical subunits. This modularity is fundamental to skeletal development and function (Wagner & Altenberg, 1996; Klingenberg, 2008), allowing distinct anatomical regions to respond to localized mechanical demands while maintaining overall structural integration. For example, the talus exhibited strong modularity, with distinct modules—head, posterior calcaneal facet, and trochlea—as confirmed by covariance ratio analysis, reflecting its role as a force distributor. The talar head articulates with the navicular for midfoot mobility and transmits body weight distally through the medial column of the foot; the trochlea participates in the ankle joint and permits dorsi‐plantarflexion; and the posterior calcaneal facet constitutes the primary subtalar joint contributing to inversion/eversion movements and experiencing peak loading during midstance. Despite this modularity in the talus, high inter‐module integration reveals that these talar regions do not develop entirely independently, balancing modular adaptation with structural coherence. Similarly, calcaneal modularity reflects functional partitioning between the sustentaculum tali (supporting the talar head and maintaining the medial longitudinal arch) and the posterior talar facet (facilitating inversion‐eversion movements).

The strong integration observed between these bone‐specific modules indicates coordinated development maintains biomechanical functionality across the entire respective bones. An equilibrium between modular independence and overall integration permits both localized optimization and the maintenance of structural integrity. During development, different regions undergo shape modifications in response to distinct loading patterns associated with specific phases of the gait cycle: the talar head adapts to forces related to forefoot push‐off and medial arch support, the trochlea responds to axial loading and rotational forces transmitted from the lower leg, while calcaneal regions differentially adapt to impact forces during heel strike and arch mechanics during midstance (DeSilva, 2009; Holowka & Lieberman, 2018). This modular architecture with high inter‐module integration represents an optimal developmental solution for accommodating complex mechanical demands on the talocalcaneal complex during the acquisition of mature bipedal locomotion.

### 4.3 Integrated internal and external hindfoot structure

Parallel analysis of trabecular architecture shows that the talus and calcaneus exhibit complementary, yet distinct, internal remodeling patterns tailored to their unique functional roles. The talus maintained relatively stable bone volume fraction and degree of anisotropy throughout development, consistent with its role as a multi‐directional force transmission hub experiencing complex, variable loading from multiple articular surfaces. In contrast, the calcaneus showed progressive increases in bone volume fraction, trabecular thickness, and higher anisotropy values with age, reflecting increasing alignment of trabecular architecture along primary loading axes. This specialized adaptation of the calcaneus may reflect its role in absorbing repetitive impact forces during initial contact. These differential trabecular patterns align with the observed greater allometric effect in calcaneal external shape compared to the talus, suggesting coordinated internal and external structural responses to increasing body mass and loading magnitudes during growth. Differential patterns observed in the talus and calcaneus in the present study corroborate previous trabecular analyses (Saers et al., 2020, 2022; Tsegai et al., 2018) that documented complementary developmental trajectories in these bones.

Our findings suggest that hindfoot development operates as an integrated system, with coordinated changes in external morphology, internal trabecular architecture, and functional behavior. The critical morphological changes of the hindfoot in the present study occur between 1–3 and 3–6 years, coinciding temporally with trabecular remodeling—from dense networks of thin trabeculae to sparser networks of thicker trabeculae—and with key locomotor milestones such as the adoption of adult‐like knee flexion patterns and functional maturation of the longitudinal arch (Bertsch et al., 2004; Sutherland et al., 1980; Zeininger et al., 2018). This correspondence in developmental timing suggests that hindfoot trabecular development is molded by biomechanical feedback from emerging locomotor behaviors rather than following a purely genetically predetermined trajectory. This finding is consistent with recent evidence that trabecular ontogeny tracks neuromuscular development and locomotor milestone acquisition (Saers et al., 2022).

The strong morphological integration documented here encompasses both external shape and internal trabecular architecture, indicating that developmental coordination operates across multiple structural scales to maintain subtalar joint congruence and functional efficiency throughout the dynamic period of locomotor acquisition. Trabecular reorganization in the calcaneus becomes particularly pronounced following the emergence of effective heel‐strike patterns between 18 and 24 months (Bertsch et al., 2004; Sutherland et al., 1980), as repetitive impact loading at initial contact becomes a dominant feature of each gait cycle.

Fossil evidence from early hominins shows mosaic evolution in hindfoot morphology, with some species exhibiting more human‐like calcaneal morphology but more ape‐like talar features, or vice versa (DeSilva et al., 2019, p. 201; Holowka & Lieberman, 2018; Zipfel et al., 2011; DeSilva et al., 2013). Trabecular analyses of fossil tali and the calcaneus similarly reveal mosaic patterns reflecting varied locomotor behaviors (Su & Carlson, 2017; Zeininger et al., 2016). Future comparative studies examining developmental integration patterns in non‐human primates and fossil hominins could test whether the degree of talus–calcaneus integration correlates with locomotor specialization (e.g., habitual arboreality, obligate terrestrial bipedalism, or knuckle‐walking quadrupedalism), potentially providing insights into the emergence of human‐like hindfoot developmental coordination in our evolutionary lineage.

### 4.4 Limitations

Several limitations should be acknowledged. The sample size, particularly in the 0–1‐year and 3–6‐year age groups, limits statistical power for detecting subtle age‐specific effects and precludes robust within‐group analyses. The small sample size of the youngest age class also limits our ability to capture the full range of variation expressed during the critical transition from pre‐walking to early walking stages. Differential trabecular bone preservation resulted in fewer calcanei being available for microstructural analysis compared to tali, potentially affecting the representativeness of trabecular developmental patterns observed in the calcaneus. Accordingly, the trabecular component of the study should be interpreted as exploratory, given the limited and uneven preservation of microstructural data across age classes. However, the observed trends remain consistent across the available age range, suggesting they represent genuine developmental signals rather than sampling artifacts.

The cross‐sectional design of the study precludes assessment of individual developmental trajectories and limits our ability to distinguish maturational changes from inter‐individual variation. Longitudinal studies tracking individuals throughout development would provide more definitive evidence for establishing ontogenetic patterns and clarifying the timing and tempo of observed morphological changes. The inclusion of specimens from different chronological periods and populations, combined with small sample sizes, may have introduced potential confounding factors including population‐specific growth patterns, environmental influences on development, and differences in footwear use or habitual activity patterns. Future studies with larger, temporally and geographically stratified samples would help clarify the relative contributions of these factors.

Finally, our analyses focus on morphological integration and cannot directly assess the genetic, developmental, or functional mechanisms underlying the observed covariation patterns. Future studies integrating geometric morphometrics with developmental genetic analyses (to identify regulatory pathways coordinating talocalcaneal growth), biomechanical modeling (to quantify loading patterns driving morphological integration), and longitudinal developmental studies would provide deeper insights into the mechanistic basis of talus–calcaneus integration and the relative roles of genetic canalization versus mechanical feedback in producing coordinated hindfoot development. Despite these limitations, our findings provide robust evidence for strong morphological integration and modular organization within the developing hindfoot skeletal elements, which have clear implications for understanding locomotor development and evolution.

## 5 CONCLUSIONS

This study provides the first quantitative assessment of morphological integration and modular organization within the human hindfoot during postnatal ontogeny.

Our findings reveal that the talus and calcaneus develop as a highly integrated biomechanical unit throughout childhood, with coordinated changes across external morphology and internal trabecular architecture coinciding with major locomotor milestones. This correspondence in developmental timing suggests that hindfoot ontogeny is influenced by biomechanical feedback from emerging locomotor behaviors. Despite strong integration, both bones exhibit modular organization, allowing functionally distinct regions to adapt semi‐independently while maintaining structural coordination.

From an evolutionary perspective, the exceptional integration documented here represents a critical adaptation for human obligate bipedalism. Fossil evidence shows mosaic hindfoot morphology in early hominins, suggesting that different integration patterns may reflect alternative locomotor strategies in extinct taxa. Clinically, these findings emphasize that hindfoot development should be assessed as an integrated functional complex, with the critical developmental window during early childhood potentially optimal for early intervention in cases of abnormal development.

In conclusion, the talus–calcaneus complex exemplifies how skeletal elements function as integrated developmental systems, providing crucial foundations for understanding how developmental constraints and mechanical demands shaped the evolution of human bipedal locomotion.

## AUTHOR CONTRIBUTIONS

**Carla Figus:** Conceptualization; Methodology; Data curation; Formal analysis; Investigation; Visualization; Writing – original draft. **Rita Sorrentino:** Resources; Writing – review & editing. **Francesca Seghi:** Visualization; Writing – review & editing. **Maria Giovanna Belcastro:** Resources; Writing – review & editing. **Kristian J. Carlson:** Supervision; Writing – review & editing.

## Supporting information


Appendix S1.

## Data Availability

The data that support the findings of this study are available from the corresponding author upon reasonable request.
