# Supplementary material for: Growing together: Developmental integration and modularity in the human talus–calcaneus complex
Source: J Anat. 2026 Jun 25:10.1111/joa.70186. Online ahead of print. doi: 10.1111/joa.70186 (PMC13398836; doi:10.1111/joa.70186)
Supplement: Supplementary file 1 — Appendix S1. [file JOA-9999-0-s001.docx]

**Supplementary Information**

**Growing Together: Developmental Integration and Modularity in the Human Talus-Calcaneus Complex**

**Carla Figus^1^*******, Rita Sorrentino^2^, Francesca Seghi^1^, Maria Giovanna Belcastro^2^, Kristian Carlson^3,4^**

**Table S1.** Complete specimen inventory and data availability. M = male; F = female; U = undetermined/unknown. Age estimated from osteological analyses (dental development and long bone metrics). All individuals included in morphometric analyses had both the talus and calcaneus preserved and suitable for landmarking. Trabecular analysis availability reflects preservation quality sufficient for micro-CT quantification (voxel resolution = 12-38 µm). Bologna specimens: documented collection with archival records (Belcastro et al., 2017); Velia specimens: Imperial Roman period skeletal series (Cipriani et al., 2020; Torino et al., 2018).

| Individual ID | Collection | Age (years) | Age Group | Sex | Morphometric Analysis | Trabecular Analysis (Talus) | Trabecular Analysis (Calcaneus) |
| --- | --- | --- | --- | --- | --- | --- | --- |
| VeliaT398 | Velia | 0.58 | 0-1 | U | Yes | No | No |
| BO58-M | Bologna | 0.92 | 0-1 | M | Yes | Yes | Yes |
| BO60-F | Bologna | 0.92 | 0-1 | F | Yes | Yes | Yes |
| VeliaT415 | Velia | 1.25 | 1-3 | U | Yes | Yes | No |
| VeliaT434 | Velia | 1.25 | 1-3 | U | Yes | Yes | No |
| BO14-M | Bologna | 1.42 | 1-3 | M | Yes | Yes | Yes |
| BO14-F | Bologna | 1.75 | 1-3 | F | Yes | Yes | Yes |
| BO7-M | Bologna | 2.75 | 1-3 | M | Yes | Yes | Yes |
| VeliaT411 | Velia | 2.83 | 1-3 | U | Yes | Yes | No |
| BO48-F | Bologna | 3.00 | 1-3 | F | Yes | Yes | Yes |
| VeliaT342 | Velia | 4.00 | 3-6 | U | Yes | Yes | No |
| BO4-F | Bologna | 5.00 | 3-6 | F | Yes | Yes | Yes |
| BO5-F | Bologna | 5.00 | 3-6 | F | Yes | Yes | Yes |
| PARMA7-F | Bologna | 5.00 | 3-6 | F | Yes | Yes | Yes |
| VeliaT375 | Velia | 5.00 | 3-6 | U | Yes | Yes | No |
| BO1-M | Bologna | 5.67 | 6-10 | M | Yes | Yes | Yes |
| BO6-F | Bologna | 5.83 | 6-10 | F | Yes | Yes | No |
| VeliaT390 | Velia | 5.75 | 6-10 | U | Yes | Yes | No |
| BO11-F | Bologna | 6.00 | 6-10 | F | Yes | Yes | Yes |
| VeliaT333 | Velia | 6.50 | 6-10 | U | Yes | Yes | Yes |
| BO6-M | Bologna | 7.00 | 6-10 | M | Yes | Yes | Yes |
| BO40-M | Bologna | 9.00 | 6-10 | M | Yes | Yes | Yes |
| VeliaT138 | Velia | 9.50 | 6-10 | U | Yes | Yes | Yes |

*Table S2. BV/TV and DA by individual*

| Individual | Age (years) | BV/TV (%) - Talus | DA - Talus | BV/TV (%) - Calcaneus | DA - Calcaneus |
| --- | --- | --- | --- | --- | --- |
| VeliaT398 | 0.58 | NA | NA | NA | NA |
| BO58-M | 0.92 | 23.24 | 0.16 | 14.09 | 0.28 |
| BO60-F | 0.92 | 20.12 | 0.26 | 14.67 | 0.38 |
| VeliaT415 | 1.25 | 20.66 | 0.13 | NA | NA |
| VeliaT434 | 1.25 | 18.60 | 0.27 | NA | NA |
| BO14-M | 1.42 | 11.80 | 0.23 | 11.87 | 0.33 |
| BO14-F | 1.75 | 13.82 | 0.26 | 14.79 | 0.30 |
| BO7-M | 2.75 | 16.36 | 0.25 | 15.51 | 0.36 |
| VeliaT411 | 2.83 | 18.39 | 0.20 | NA | NA |
| BO48-F | 3.00 | 15.24 | 0.25 | 14.77 | 0.34 |
| VeliaT342 | 4.00 | 15.82 | 0.22 | NA | NA |
| BO4-F | 5.00 | 16.22 | 0.22 | 16.62 | 0.31 |
| BO5-F | 5.00 | 19.61 | 0.22 | 14.16 | 0.29 |
| PARMA7-F | 5.00 | 22.41 | 0.26 | 17.99 | 0.28 |
| VeliaT375 | 5.00 | 17.68 | 0.21 | NA | NA |
| BO1-M | 5.67 | 16.94 | 0.22 | 16.58 | 0.31 |
| BO6-F | 5.83 | 17.68 | 0.21 | NA | NA |
| VeliaT390 | 5.75 | 15.18 | 0.22 | NA | NA |
| BO11-F | 6.00 | 17.55 | 0.21 | 16.55 | 0.32 |
| VeliaT333 | 6.50 | 22.81 | 0.23 | 20.35 | 0.25 |
| BO6-M | 7.00 | 13.36 | 0.22 | 18.83 | 0.30 |
| BO40-M | 9.00 | 13.36 | 0.22 | 16.26 | 0.36 |
| VeliaT138 | 9.50 | 34.78 | 0.18 | 20.40 | 0.26 |


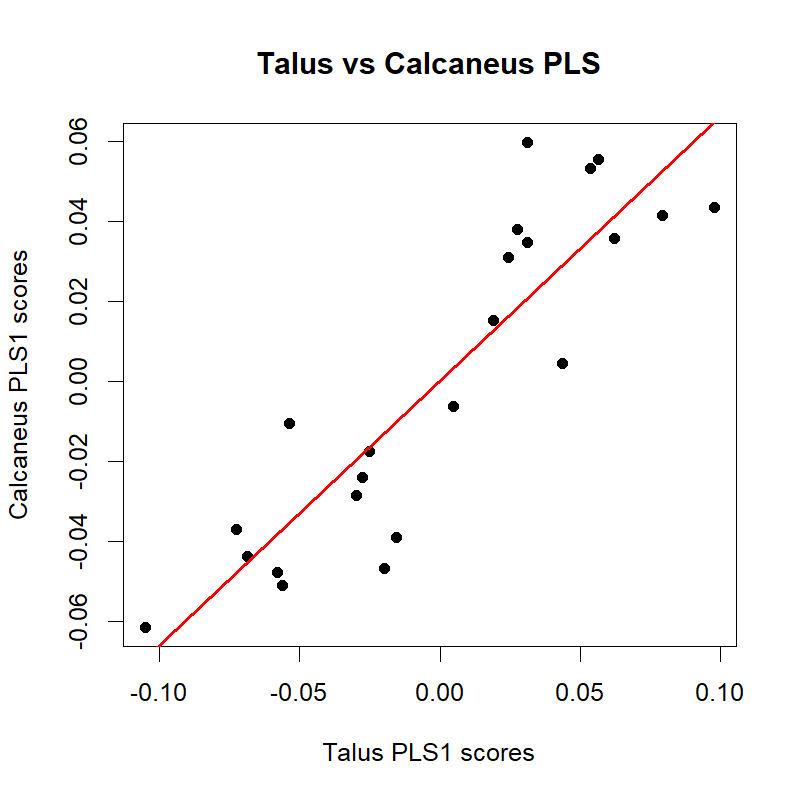


Figure S1. Partial Least Squares (PLS) analysis of shape covariation between talus and calcaneus. Each point represents an individual specimen. The first PLS axis (PLS1) captures the primary pattern of covariation between the two bones, with the regression line indicating a strong positive relationship. Specimens with positive PLS1 scores exhibit coordinated shape changes in both talus and calcaneus.


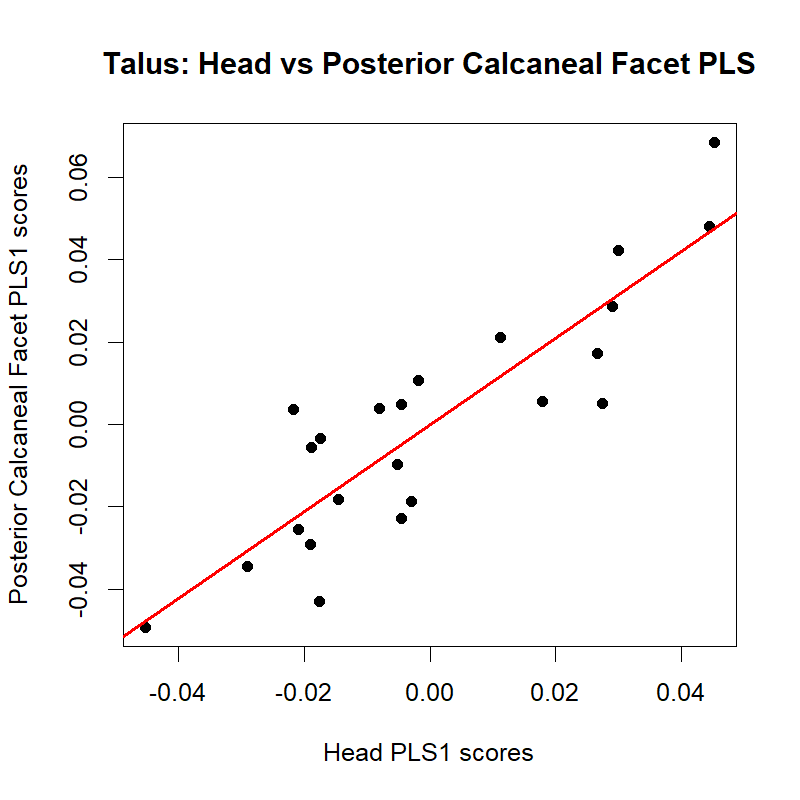

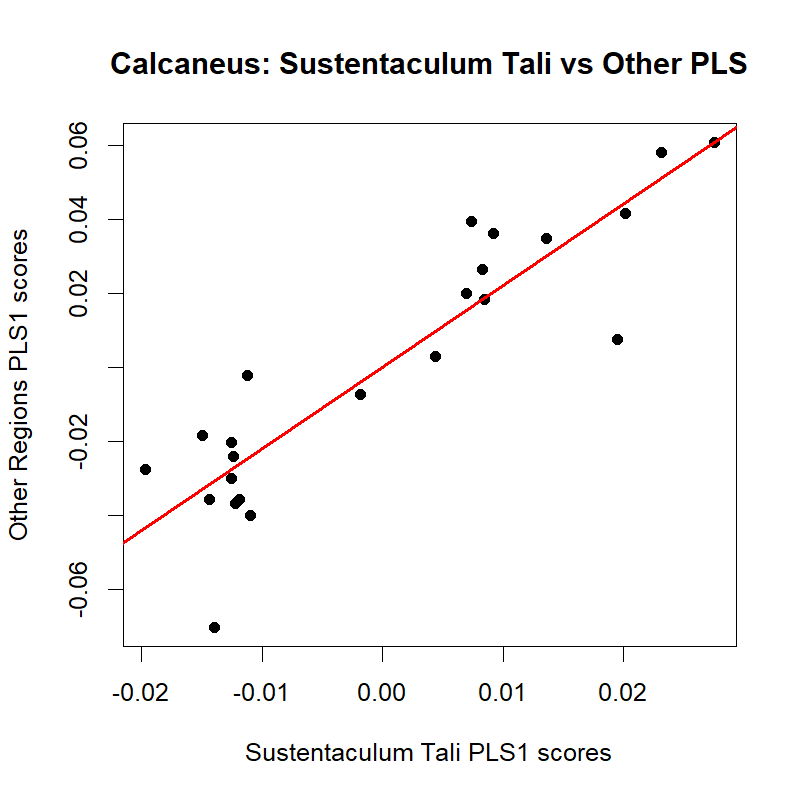

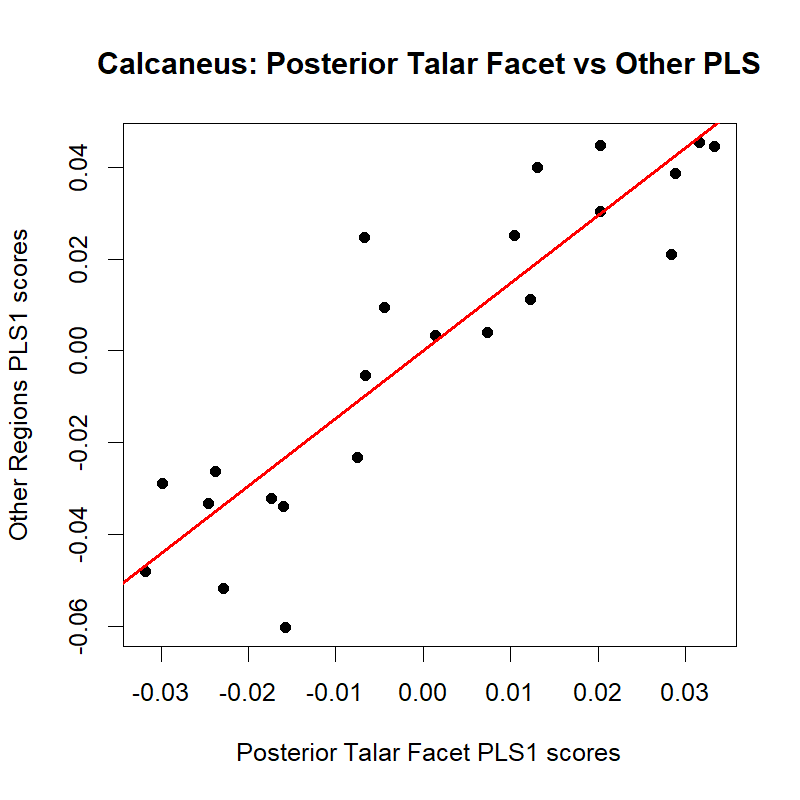

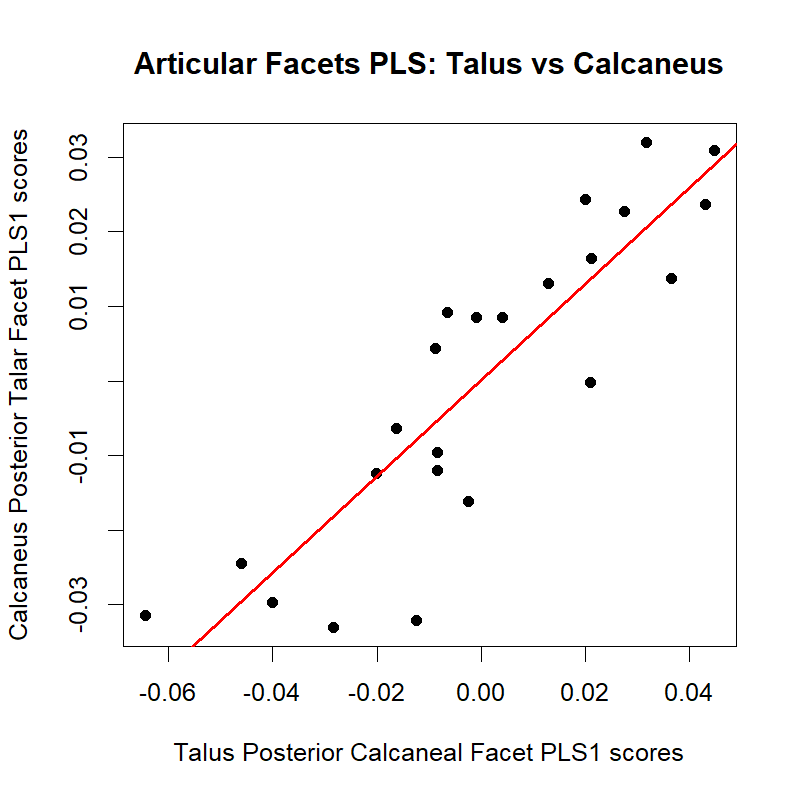

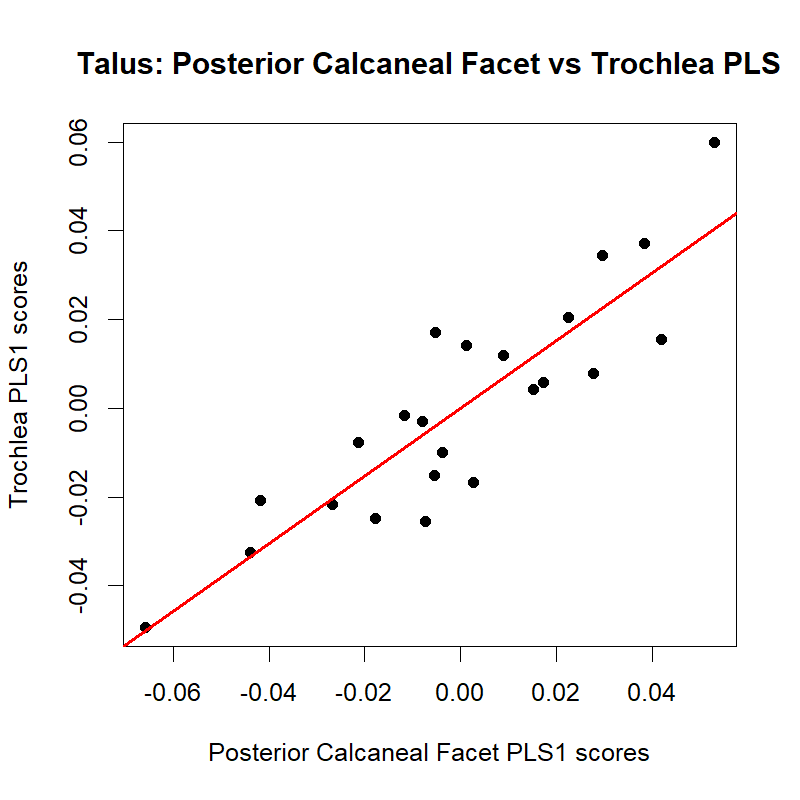

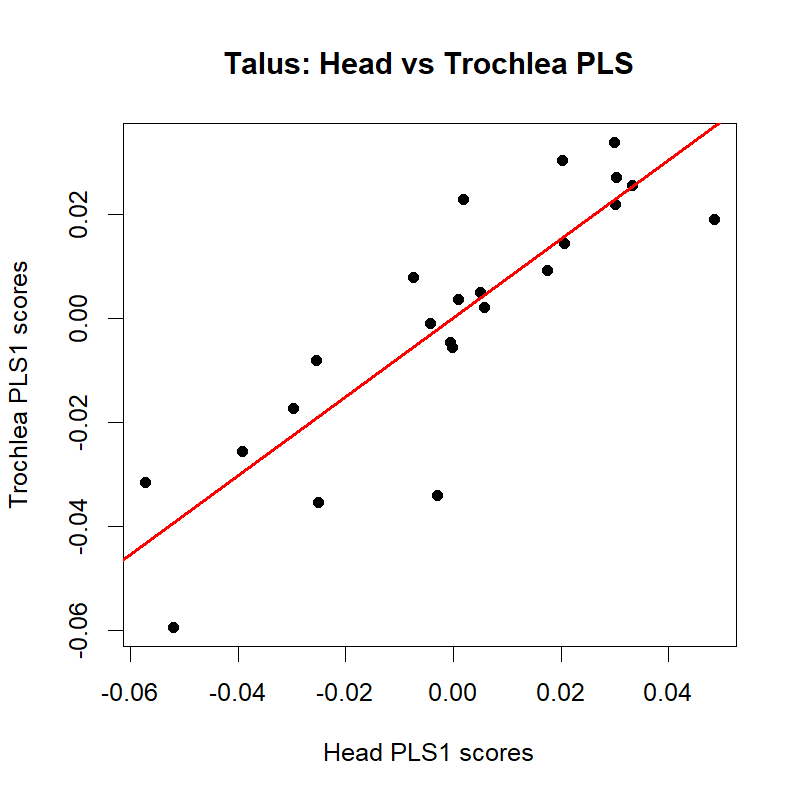


*Figure S2. Morphological integration patterns in the tarsus revealed through Partial Least Squares (PLS) analyses. Six bivariate plots show covariation between: (top row) talus head vs posterior calcaneal facet, calcaneus sustentaculum tali vs other regions, and calcaneus posterior talar facet vs other regions; (bottom row) articular facets between talus and calcaneus, talus posterior calcaneal facet vs trochlea, and talus head vs trochlea. Each point represents an individual specimen, and red lines indicate the regression of PLS1 scores, demonstrating coordinated shape variation across anatomically and functionally related regions.*
